# PIFs- and COP1-HY5-mediated temperature signaling in higher plants

**DOI:** 10.1007/s44154-022-00059-w

**Published:** 2022-08-30

**Authors:** Yeting Bian, Li Chu, Huan Lin, Yaoyao Qi, Zheng Fang, Dongqing Xu

**Affiliations:** State Key Laboratory of Crop Genetics and Germplasm Enhancement, National Center for Soybean Improvement, College of Agriculture, Nanjing Agricultural University, Nanjing, 210095 China

**Keywords:** Light signaling, Low temperature, Warm temperature, phyB, PIFs, COP1, HY5, ELF3

## Abstract

Plants have to cope with the surrounding changing environmental stimuli to optimize their physiological and developmental response throughout their entire life cycle. Light and temperature are two critical environmental cues that fluctuate greatly during day-night cycles and seasonal changes. These two external signals coordinately control the plant growth and development. Distinct spectrum of light signals are perceived by a group of wavelength-specific photoreceptors in plants. PIFs and COP1-HY5 are two predominant signaling hubs that control the expression of a large number of light-responsive genes and subsequent light-mediated development in plants. In parallel, plants also transmit low or warm temperature signals to these two regulatory modules that precisely modulate the responsiveness of low or warm temperatures. The core component of circadian clock ELF3 integrates signals from light and warm temperatures to regulate physiological and developmental processes in plants. In this review, we summarize and discuss recent advances and progresses on PIFs-, COP1-HY5- and ELF3-mediated light, low or warm temperature signaling, and highlight emerging insights regarding the interactions between light and low or warm temperature signal transduction pathways in the control of plant growth.

## Introduction

Light and temperature are two of the most essential environmental factors controlling plant growth and development. Both light and temperature tightly and synergistically control diverse physiological and developmental processes in plants, including seed germination, seedling development, morphogenesis, flowering, metabolism, and immunity (Shi et al. 2018; Casal and Balasubramanian 2019; Ding et al. 2020; Ding and Yang 2022; Li et al. 2022; Qi et al. 2022). As sessile organisms, plants have evolved complex molecular regulatory networks for coping with and responding appropriately to daily and seasonal fluctuations in light and temperature in order to ensure survival.

Various light signals are perceived by at least five classes of wavelength-specific photoreceptors in Arabidopsis: phytochromes (phyA-phyE) sense red and far-red light; cryptochromes (CRY1 and CRY2), phototropins (PHOT1 and PHOT2), and ZEITLUPE family members (ZTL, FKF1, and LKP2) perceive ultraviolet (UV)-A and blue light; and UV-B RESISTANCE LOCUS 8 (UVR8) absorbs UV-B (Paik and Huq 2019; Cheng et al. 2021; Podolec et al. 2021). These photoreceptors are responsible for perceiving the various light signals from the sun and then transmitting the information to downstream signaling networks, which precisely control plant growth and development (Pham et al. 2018; Wang et al. 2018; Liang et al. 2019; Podolec et al. 2021). The PHYTOCHROME INTERACTING FACTORs (PIFs) and the CONSTITUTIVELY PHOTOMORPHOGENIC 1-ELONGATED HYPOCOTYL 5 (COP1-HY5) modules act downstream of the photoreceptors to control the expression of many genes and subsequent light-controlled physiological and developmental processes in plants (Paik et al. 2017; Liang et al. 2019; Song et al. 2020; Xu 2020; Jing and Lin 2020).

Low and warm temperatures strongly affect plant growth, often leading to drastically morphological alternations at both the seedling and adult growth stages. Low temperatures inhibit root and hypocotyl elongation, adult plant morphogenesis, and initiation of floral development (Ding et al. 2019, 2020; Ding and Yang 2022). Warm temperatures (below the heat stress range) result in plant thermomorphogenesis, showing dramatically elongated roots, hypocotyls, and petioles, as well as early flowering and accelerated leaf senescence (Casal and Balasubramanian 2019; Brightbill and Sung 2022; Han et al. 2022). Cellular membranes, calcium (Ca^2+^) channels, and rice CHILLING TOLERANCE DIVERGENCE 1 (COLD1) are responsible for low temperature sensing (Ma et al. 2015; Guo et al. 2018; Zhang et al. 2019; Ding and Yang 2022). Recent studies have revealed that the red-light photoreceptor phyB, its interacting partner PIF7, and a core component of the plant circadian clock EARLY FLOWERING 3 (ELF3) function as warm temperature sensors (Legris et al. 2016; Jung et al. 2016, 2020; Chung et al. 2020; Fiorucci et al. 2020).

Multiple light signaling components are involved in temperature-mediated development, suggesting that both light and temperature signaling work in concert to control plant growth and development (Shi et al. 2018; Casal and Balasubramanian 2019; Ding et al. 2020) (Table 1). Both the PIFs and COP1-HY5, two core light signaling regulatory hubs, play critical roles in controlling the responsiveness of plants to low and warm temperatures. Taken together, it appears that both light and temperature signaling networks work in concert to optimize plant growth during daily and seasonal changes accompanied by fluctuating light and temperature signals. This review summarizes recent advances in our understanding of the coordinated regulation of light- and temperature-mediated adaptive responses in plants and offers potential directions for future studies.

**Table 1:** Summary of key components involved in the regulation of light and temperature signaling

Plant Species	Name	Gene Locus ID	Mode of action in the regulation of distinct physiological processes			Reference
			Light signaling	Cold signaling	Warm temperature signaling	
Arabidopsis (*Arabidopsis* *thaliana*)	CBF1	AT4G25490	Negative	Positive	ND	Lee and Thomashow 2012; Dong et al. 2020
	CCA1	AT2G46830	Negative	ND	Positive	Wang and Tobin 1998; Sun et al. 2019
	COP1	AT2G32950	Negative	Negative	Positive	Deng et al. 1992; Catalá et al. 2011; Delker et al. 2014
	COR27	AT5G42900	Negative	Negative	ND	Li et al. 2016; Zhu et al. 2020
	COR28	AT4G33980	Negative	Negative	ND	Li et al. 2016; Zhu et al. 2020
	CRY1	AT4G08920	Positive	ND	Negative	Casal and Boccalandro 1995; Zhou et al. 2019
	CRY2	AT1G04400	Positive	Positive	ND	Lin et al. 1998; Li et al. 2021a
	DET1	AT4G10180	Negative	ND	Positive	Chory et al. 1989; Delker et al. 2014
	EBF1	AT2G25490	Positive	Positive	ND	Dong et al. 2017; Jiang et al. 2017
	EBF2	AT5G25350	Positive	Positive	ND	Dong et al. 2017; Jiang et al. 2017
	ELF3	AT2G25930	Positive	ND	Negative	Nusinow et al. 2011; Jung et al. 2020;
	ELF4	AT2G40080	Positive	ND	Negative	Nusinow et al. 2011; Jung et al. 2020
	HFR1	AT1G02340	Positive	ND	Negative	Fairchild et al. 2000; Ikeda et al. 2021
	HLS1	AT4G37580	Negative	ND	Positive	Lehman et al. 1996; Jin et al. 2020
	HMR	AT2G34640	Positive	ND	Positive	Chen et al. 2010; Huai et al. 2018; Qiu et al. 2019
	HY5	AT5G11260	Positive	Positive	Negative	Oyama et al. 1997; Catalá et al. 2011; Kim et al. 2017
	KAI2	AT4G37470	Positive	ND	Negative	Waters et al. 2015; Park et al. 2022
	PAR1	AT2G42870	Positive	ND	Negative	Zhou et al. 2014; Ikeda et al. 2021
	PCH1	AT2G16365	Positive	ND	Negative	Enderle et al. 2017; Murcia et al. 2021
	PCHL	AT4G34550	Positive	ND	Negative	Enderle et al. 2017
	phyB	AT2G18790	Positive	Positive	Negative	Wagner et al. 1991; Jung et al. 2016; Legris et al. 2016; Jiang et al. 2020;
	PIF1	AT2G20180	Negative	Negative	Positive	Shen et al. 2005; Jiang et al. 2020; Lee et al. 2021a
	PIF3	AT1G09530	Negative	Negative	Positive	Bauer et al. 2004; Dong et al. 2017; Lee et al. 2021a
	PIF4	AT2G43010	Negative	Negative	Positive	Huq and Quail 2002; Koini et al. 2009; Jiang et al. 2020
	PIF5	AT3G59060	Negative	Negative	Positive	Khanna et al. 2004; Jiang et al. 2020; Lee et al. 2021a
	PIF7	AT3G59060	Negative	Negative	Positive	Leivar et al. 2008; Lee and Thomashow 2012; Chung et al. 2020; Fiorucci et al. 2020
	PHOT1	AT3G45780	Positive	ND	ND	Liscum and Briggs 1995
	PHOT2	AT5G58140	Positive	ND	ND	Kagawa et al. 2001
	RCB	AT4G28590	Positive	ND	Positive	Qiu et al. 2021
	SEU	AT1G43850	Negative	ND	Positive	Huai et al. 2018; Qiu et al. 2019
	SHB1	AT4G25350	Positive	ND	Positive	Kang and Ni 2006; Sun et al. 2019
	SMAX1	AT5G57710	NI	ND	Positive	Park et al. 2022
	TCP17	AT5G08070	Negative	ND	Positive	Zhou et al. 2018; Han et al. 2019; Zhou et al. 2019
	TOC1	AT5G6138	Positive	ND	Negative	Sato et al. 2002; Zhu et al. 2016
	UVR8	AT5G63860	Positive	ND	Positive	Rizzini et al. 2011; Hayes et al. 2017
	ZTL	AT5G57360	Positive	ND	Positive	Somers et al. 2004; Saitoh et al. 2021
Tomato (*Solanum lycopersicum*)	SlHY5	Solyc08g061130	Positive	Positive	ND	Zhang et al. 2020; Yang et al. 2022
	SlPIF4	Solyc07g043580	NI	Positive	Positive	Rosado et al. 2019; Wang et al. 2020
	SlphyA	Solyc10g044670	Positive	Positive	ND	Ernesto Bianchetti et al. 2018; Wang et al. 2020
	SlphyB1	Solyc01g059870	Positive	Negative	ND	Ernesto Bianchetti et al. 2018; Wang et al. 2020
	SlphyB2	Solyc05g053410	Positive	Negative	ND	Ernesto Bianchetti et al. 2018; Wang et al. 2020
Liverwort (*Marchantia polymorpha*)	MpPHOT	Mapoly0133s0008	Positive	Positive	ND	Fujii et al. 2017

*Abbreviations*: *CBF1* C-REPEAT/DRE BINDING FACTOR 1, *CCA1* CIRCADIAN CLOCK ASSOCIATED 1, *COP1* CONSTITUTIVE PHOTOMORPHOGENIC 1, *COR27* COLD REGULATED GENE 27, *COR28* COLD REGULATED GENE 28, *CRY1* CRY1, CRYPTOCHROME 1, *CRY2* CRYPTOCHROME 2, *DET1* DE-ETIOLATED 1, *EBF1* EIN3-BINDING F BOX PROTEIN 1, *EBF2* EIN3-BINDING F BOX PROTEIN 2, *ELF3* EARLY FLOWERING 3, *ELF4* EARLY FLOWERING 4, *HFR1* LONG HYPOCOTYL IN FAR-RED1, *HLS1* HOOKLESS 1, *HMR* HEMERA, *HY5* ELONGATED HYPOCOTYL 5, *KAI2* KARRIKIN INSENSITIVE 2, *PAR1* PHYTOCHROME RAPIDLY REGULATED 1, *PCH1* PHOTOPERIODIC CONTROL OF HYPOCOTYL 1, *PCHL* PCH1-LIKE, *phyA* PHYTOCHROME A, *phyB* PHYTOCHROME B, *PIF1* PHYTOCHROME INTERACTING FACTOR 1, *PIF3* PHYTOCHROME INTERACTING FACTOR 3, *PIF4* PHYTOCHROME INTERACTING FACTOR 4, *PIF5* PHYTOCHROME INTERACTING FACTOR5, *PIF7* PHYTOCHROME INTERACTING FACTOR 7, *PHOT1* PHOTOTROPIN 1, *PHOT2* PHOTOTROPIN2, *RCB* REGULATOR OF CHLOROPLAST BIOGENESIS, *SEU* SEUSS, *SHB1* SHORT HYPOCOTYL UNDER BLUE1, *SMAX1* SUPPRESSOR OF MAX2 1, *TCP17* TEOSINTE BRANCHED 1/CYCLOIDEA/PCF 17, *TOC1* TIMING OF CAB2 EXPRESSION 1, *UVR8* UV-B RESISTANCE LOCUS 8, *ZTL* ZEITLUPE, *Sl Solanum lycopersicum*, *Mp Marchantia polymorpha*, *ND* Not Determined, *NI* Not Involved

## phys sense and respond to temperature

The red and far-red light photoreceptors phys are required and essential for low temperature response (Kim et al. 2002; Franklin and Whitelam 2007; Jiang et al. 2020). Nuclear-localized phyB at 4 °C or 16 °C is abundant in comparison with that at 22 °C, suggesting that low temperatures promote the accumulation of phyB in the nucleus (Jiang et al. 2020; Lee et al. 2022). Considering that Pfr form of phyB mainly resides within the nucleus (Pham et al. 2018; Cheng et al. 2021), thus low temperatures might trigger the Pr form of phyB to convert to Pfr state. However, this hypothesis requires additional experimental validation. phyB sense the temperature signals in a broad range from 10 °C to 30 °C through a Pfr to Pr thermoconversion mechanism (Jung et al. 2016; Legris et al. 2016; Sellaro et al. 2019). Red light-excited phyA and phyB inactivate the COP1 activity and facilitate the degradation of PIFs at optimal growth temperatures for Arabidopsis (i.e. 22 °C) to promote phototmorphogenesis (Hoecker 2017; Pham et al. 2018; Podolec and Ulm 2018; Han et al. 2020). Dark, shade, far-red light and warm temperatures (i.e. 28 °C to 30 °C) redundantly trigger a large portion of biologically active phyB-Pfr form to convert back to inactive Pr state, thus allowing the accumulation of growth-promoting factor like PIF4 (Jung et al. 2016; Legris et al. 2016; Han et al. 2020; Cheng et al. 2021). Photo-excited phyB proteins spontaneously form liquid-like droplets. Its N-terminal extension directly senses the warm temperature signals to modulate the phase behavior of phyB droplets (Chen et al. 2022). Thus, interweaved light and temperature signals coordinately and synergistically controls the pool of phyB Pfr form and its liquid-like condensates, through which these two environmental signals precisely regulate the downstream signaling pathways to ensure an optimized plant growth and development. The blue light photoreceptor PHOT in the liverwort *Marchantia polymorpha* senses the cold temperatures through its light/oxygen/voltage (LOV) domains. The lifetimes of its photoactivated chromophores are altered at distinct temperatures (Fujii et al. 2017). Very recent studies have documented that the photoreceptors CRYs, PHOTs, ZTL and UVR8 in Arabidopsis play critical roles in the low or warm temperature-regulated root, hypocotyl growth and/or flowering (Ma et al. 2016; Hayes et al. 2017; Zhou et al. 2019; Li et al. 2021a; Saitoh et al. 2021), however, whether these photoreceptors act as temperature sensors remains to be explored.

## PIFs-mediated cold stress

Low temperature is arguably one of the key environmental factors that affect the plant growth and development. Plants have evolved sophisticated regulatory mechanisms to adapt to low temperatures such as cold accumulation (Ding et al. 2020; Ding and Yang 2022). PIFs signaling has been shown to be essential for the appropriate responsiveness of low temperatures (Jiang et al. 2017, 2020). Under long-day conditions, phyB and its signaling components PIF4 and PIF7 negatively control the transcription of *C-REPEAT BINDING FACTORs* (*CBFs: CBF1, CBF2* and *CBF3*), which are key regulators of cold signaling (Lee and Thomashow 2012; Liu et al. 2018). In the light, the E3 ubiquitin ligases EIN3-BINDING F-BOX PROTEIN 1 (EBF1) and EBF2 target PIF3 for ubiquitination and degradation (Dong et al. 2017; Jiang et al. 2017). Low temperatures promote the degradation of EBF1 and EBF2, allowing the accumulation of PIF3 under cold stress. Increased PIF3 directly associates with the promoter regions of *CBFs* to inhibit their expression, thereby negatively mediating plant freezing tolerance (Jiang et al. 2017). In response to low temperature, CBF proteins inhibit the concurrent degradation of PIF3 and phyB by interacting with PIF3. Cold-stabilized phyB facilitates the degradation of PIF1, PIF4, and PIF5, all of which negatively regulate cold stress, thus leading to the increased plant freezing tolerance (Jiang et al. 2020). These findings reveal a feedback signaling circuitry consisting of CBFs, phyB and PIFs that integrate light and cold signaling pathways in plants. In addition, CBF1 not only interferes with the association of phyA or phyB with PIF4 and PIF5, but also directly binds to the *PIF4* and *PIF5* promoters to activate their transcription. Thus, these molecular events lead to the high abundance of PIF4 and PIF5 and promotion of hypocotyl growth (Dong et al. 2020). Notably, CBF1 promotes the accumulation of PIF4 and PIF5 at optimal growth temperatures for Arabidopsis (17 °C or 22 °C), but not at low temperature (4 °C) conditions. These facts suggest that CBF1 mediates different physiological and developmental responses in a temperature dependent manner. CBF2 and CBF3, the two close members of CBF1, are likely not involved in light signaling (Dong et al. 2020), suggesting that these three CBFs have distinct functions in the regulation of plant growth and development.

In tomato, far-red light and SlphyA positively control the abundance of SlPIF4, which directly binds to the *SlCBFs* promoter regions to activate their expression and increase cold tolerance (Wang et al. 2020). Contrarily, SlPIF4 negatively regulates cold tolerance in tomato anthers through SlDYT1-SlTDF1-triggered tapetum development and tapetal programmed cell death (Pan et al. 2021), suggesting that SIPIF4 exerts distinct and specific functions in different tissues or organs under low temperatures. In summary, phys, PIFs and CBFs form a complex molecular network at both the transcriptional and post-translational levels to orchestrate plant growth and development in response to cold stress in the light.

## COP1-HY5 signaling-mediated cold stress

**Fig. 1:**
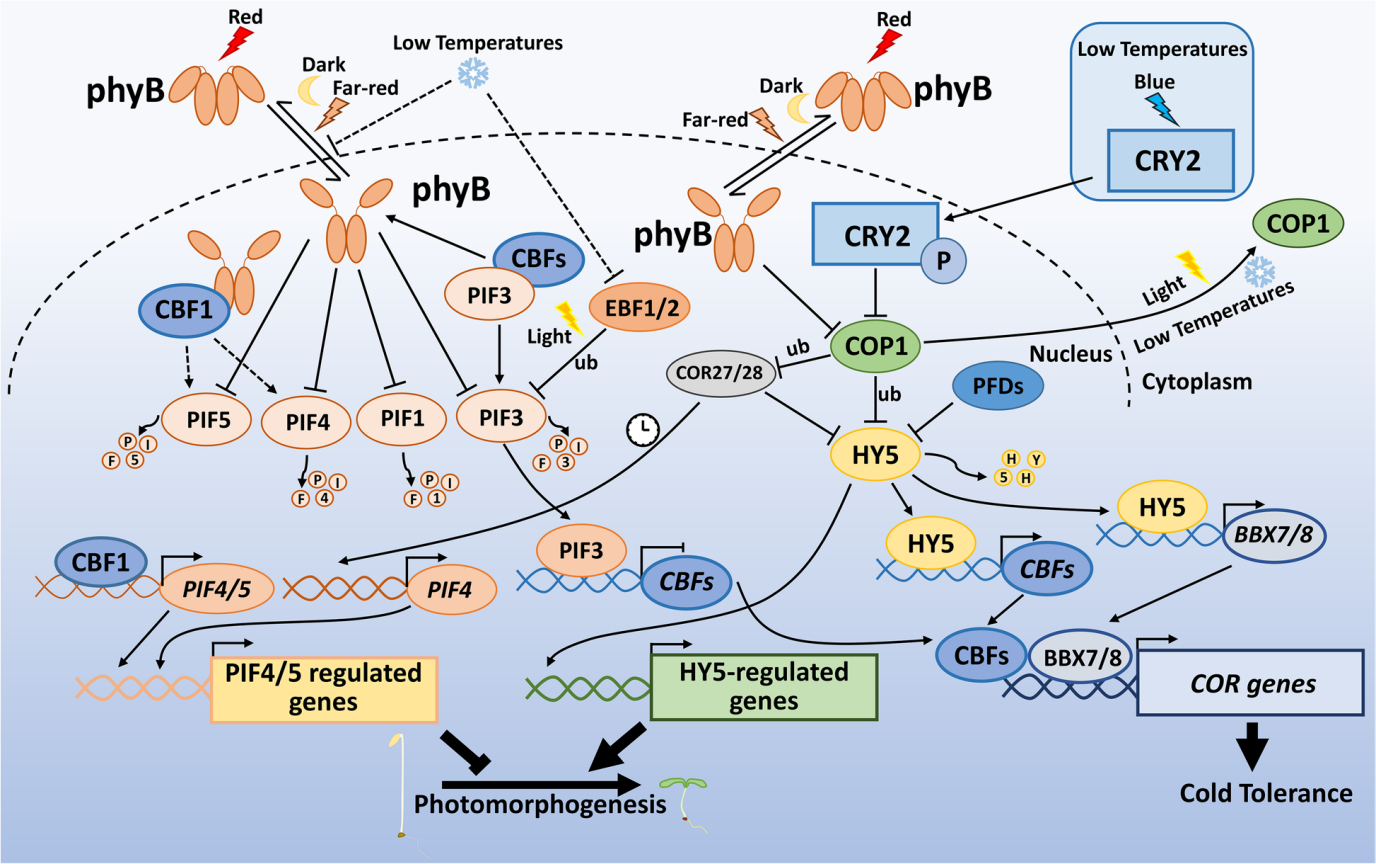
PIFs and COP1-HY5 regulatory modules integrate light and low temperature signaling. Low temperatures negatively affect the light-triggered degradation of PIF3 by EBF1/2, allowing the accumulation of PIF3 that directly binds to the promoters of *CBF* genes and represses their expression. Accumulated CBFs interact with PIF3 to prevent concurrent degradation of PIF3 and phyB. Cold-stabilized phyB promotes the degradation of PIF1, PIF4, and PIF5. Upon red light irradiation, CBF1 interacts with phyB and promotes the accumulation of PIF4 and PIF5 at both transcriptional and protein levels. Cold- and blue light-induced COR27 and COR28 up-regulate the *PIF4* expression in a circadian clock dependent manner. PIF4 and PIF5 regulate downstream target genes’ expression to repress photomorphogenesis. Light and low temperatures trigger the COP1 translocate from the nucleus to the cytoplasm. Low temperatures stabilize blue light-induced phosphorylated CRY2 that interacts with COP1 to compete with HY5. Consequently, these events lead to the accumulation of HY5. Accumulated HY5 activates the transcription of *BBX7* and *BBX8* to positively regulate freezing tolerance by controlling a set of *COR* genes’ expression. In parallel, HY5 regulates the expression of numerous target genes to promote photomorphogenesis. ub indicates ubiquitination

In darkness, COP1 targets HY5 for ubiquitination and degradation, while light inactivates COP1, leading to the accumulation of HY5, allowing the COP1-HY5 module to precisely regulate the plant response to the transition from day to night (Osterlund et al. 2000; Hoecker 2017; Podolec and Ulm 2018; Han et al. 2020). Additionally, the COP1-HY5 regulatory hub has been documented to play critical roles in promoting plant freezing tolerance (Catalá et al. 2011) (Fig. 1). Cold triggers the translocation of COP1 from the nucleus to the cytoplasm, thus resulting in an abundance of HY5, which activates *CBF1* and a number of cold-responsive genes’ expression (Catalá et al. 2011). Low temperatures also induce the accumulation of PREFOLDINs (PFDs) in the nucleus, where they interact with HY5 and promote its ubiquitination and degradation independently of COP1 (Perea-Resa et al. 2017). These distinct regulatory mechanisms for controlling HY5 abundance ensure that plants respond optimally to cold stress. Blue light also increases cold tolerance by way of the COP1-HY5 module in plants. Specifically, cold stress stabilizes the blue light-triggered phosphorylated form of CRY2, which competes with HY5 to disrupt the COP1-HY5 interaction, leading to the accumulation of HY5. Accumulated HY5 directly promotes the transcription of *B-BOX PROTEIN 7* (*BBX7*) and *BBX8*, which regulate the expression of a group of cold-responsive genes to increase plant freezing tolerance (Li et al. 2021a). Blue light and low temperatures also induce the accumulation of COLD REGULATED 27 (COR27) and COR28, both of which negatively control freezing tolerance in plants (Li et al. 2016). In darkness, COP1 promotes the degradation of COR27 and COR28 (Li et al. 2020a; Kahle et al. 2020; Zhu et al. 2020), while in the light, accumulated COR27 and COR28 not only repress HY5 transcriptional activation activity, but also up-regulate *PIF4* expression in a circadian clock-dependent manner (Li et al. 2020a; Zhu et al. 2020). As a consequence, COR27 and COR28 integrate light, temperature, and circadian clock signaling to modulate the development in plants (Li et al. 2016, 2020a; Wang et al. 2017; Kahle et al. 2020; Zhu et al. 2020). These documented molecular events suggest that various light and low temperature signals transmitted from the photoreceptors converge on COP1-HY5 hub, at least in part, governing downstream singling pathways that enable plants to appropriately respond to cold stress and changing light signals.

In tomato, cold stress triggers the transcription of *SlHY5*, *SlMYB15*, and *SlCBFs*. SlHY5 directly binds to the promoter region of *SlMYB15* to activate its expression, and both SlHY5 and SlMYB15 up-regulate *SlCBF1*, *SlCBF2*, and *SlCBF3* expression to increase cold tolerance (Zhang et al. 2020). In blood orange, under cold conditions, CsHY5 directly binds to the *G-box cis*-element of the *CsRuby1* promoter, a key activator of anthocyanin biosynthesis, to activate its expression, thereby leading to the accumulation of anthocyanin in the fruit peel (Huang et al. 2019). Thus, light and low temperature signals are integrated at the molecular level to optimize plant physiological response to cold stress. It appears that multiple components of both the light and low temperature signaling networks like HY5 and CBFs act coordinately and synergistically to maintain proper expression patterns of light- and cold-responsive genes, which in turn help plants to appropriately respond and adapt to fluctuating light and temperatures in natural conditions.

## PIFs-mediated warm temperature response

Warm temperatures have profound impacts on plant growth and development. In plant cells, the red-light photoreceptor phyB exists as two interconvertible isoforms, the bioactive Pfr form and bioinactive Pr form. Red light triggers the conversion of phyB from inactive Pr form to biologically active Pfr isoform and then translocates from the cytoplasm to the nucleus, where Pfr form of phyB directly interacts with PIF4 and promotes its phosphorylation, poly-ubiquitination, and protein turnover via the 26S proteasome system at optimal growth temperatures (Pham et al. 2018; Cheng et al. 2021). Warm temperatures trigger Pfr to convert to the inactive Pr form, allowing the accumulation of PIF4 and promoting plant growth (Jung et al. 2016; Legris et al. 2016). The phyB-PIF4 module represents a central regulatory hub which integrates light and warm temperature signals to modulate multiple physiological and developmental progresses in plants (Shi and Zhu 2021; Li et al. 2022; Qi et al. 2022). A recent study showed that overexpression of epidermal *phyB* suppresses plant thermal response. Moreover, epidermis-specific expression of *PIF4*, but not vasculature-specific expression of *PIF4*, promotes the transcription of auxin biosynthesis- and signaling-related genes and the elongation of hypocotyls at warm ambient temperatures (Kim et al. 2020), suggesting that phyB-PIF4 promotes thermomorphogenesis in a tissue-dependent manner. phyB senses the ambient temperature under diurnal light-dark conditions (Qiu et al. 2019; Murcia et al. 2021). phyB activates the thermosensory response by regulating the PIF4 stability and activity during the daytime (Qiu et al. 2019). At night, phyB stores nighttime temperature signals to regulate hypocotyl growth during the subsequent photoperiod (Murcia et al. 2021). Light and warm temperatures have opposite effects on phyB Pfr-to-Pr interconversion, how these two external cues precisely maintain the appropriate pool of phyB-Pfr in regulating of light-mediated thermomorphogenesis during the day-night cycles awaits further detailed investigation.

As a core signaling node, multiple regulators converge on phyB-PIF4 to modulate its action at the transcriptional, post-transcriptional, and post-translational levels in response to light and warm temperatures. At warm temperatures, SUPRESSOR OF phyA-105 (SPA) proteins destabilize phyB while stabilizing PIF4. SPA1 is able to phosphorylate PIF4 in vitro, implying that the SPA1 kinase activity is necessary for PIF4 activity and PIF4-mediated thermomorphogenesis (Lee et al. 2020). SUPPRESSOR OF MAX2 1 (SMAX1) associates with phyB and negatively affects its repression on PIF4 function, thus promoting the hypocotyl thermomorphogenesis (Park et al. 2022). HOOKLESS1 (HLS1) interacts with PIF4 to co-regulate a set of transcriptional and post-transcriptional events to promote thermomorphogenesis (Jin et al. 2020). Additionally, SEUSS (SEU) and HEMERA (HMR) directly associate with PIF4 to enhance its transcriptional activation activity towards target genes, thus leading to the promotion of thermomorphogenesis (Huai et al. 2018; Qiu et al. 2019). Photoactivated phys stabilize HMR that promotes the degradation of PIF1 and PIF3 at the optimal growth temperature for Arabidopsis (Chen et al. 2010; Galvão et al. 2012; Qiu et al. 2015). At warm temperatures, REGULATOR OF CHLOROPLAST BIOGENESIS (RCB) interacts and functions collaboratively with HMR to stabilize PIF4 and trigger thermomorphogenesis (Qiu et al. 2021). TEOSINTE BRANCHED 1/CYCLOIDEA/PCF 5 (TCP5), TCP13, and TCP17 not only physically interact with PIF4 to enhance its transcriptional activity, but also directly associate with its promoter to activate its transcription at warm temperatures (Han et al. 2019; Zhou et al. 2019). In addition, *SHORT HYPOCOTYL UNDER BLUE 1* (SHB1) and CIRCADIAN CLOCK ASSOCIATED 1 (CCA1) up-regulate *PIF4* expression in both a red-light- and circadian clock-dependent manner to trigger thermomorphogenesis in response to elevated ambient temperatures (Sun et al. 2019). Evening-expressed clock component TIMING OF CAB2 EXPRESSION 1 (TOC1) directly inhibits PIF4 activity, thereby repressing thermomorphogenesis in the evening (Zhu et al. 2016). Taken together, these interactions suggest that CCA1 and TOC1 mediate the circadian gating of thermomorphogenic response by modulating the action of PIF4. The INO80 CHROMATIN REMODELING COMPLEX (INO80-C) interacts with PIF4 to regulate H2A.Z eviction at PIF4 targets, which is mediated by the transcription elongation factors SPT4 and SPT5 at elevated temperatures (Xue et al. 2021). Warm temperatures trigger the stabilization of the chromatin-modifying enzyme HISTONE DEACETYLASE 9 (HDA9), which permits the net eviction of the H2A.Z histone variant from nucleosomes associated with *YUCCA8* (*YUC8*). This modulation facilitates the binding of PIF4 to *YUC8*, and subsequently activates auxin biogenesis as well as thermomorphogenesis at warm temperatures (van der Woude et al. 2019).

In addition, blue and UV-B light mediates the plant thermal response through modulating the action of PIF4 as well. Blue light inhibits warm temperature-prompted hypocotyl elongation via CRY1 and its downstream signaling components. Upon exposure to blue light and warm temperatures, CRY1 interacts directly with PIF4 to repress the transcription of target genes involved in auxin biosynthesis (Ma et al. 2016). CRY1 represses the activity of TCP17 at ambient temperatures, but elevated temperatures stabilize TCP17 and release it from the CRY1-TCP17 complex, leading to the up-regulation of *PIF4* (Zhou et al. 2019). PIF4 directly associates with the promoter of *CRYPTOCHROME-INTERACTING BASIC HELIX-LOOP-HELIX 1* (*CIB1*) to activate its expression. PHYTOCHROME RAPIDLY REGULATED1 (PAR1) and LONG HYPOCOTYL IN FAR-RED1 (HFR1) disrupt the binding of PIF4 to the *CIB1* promoter. In addition, PAR1 negatively regulates the DNA binding activity of CIB1 towards target genes, which promote cell elongation and hypocotyl growth at warm temperatures (Ikeda et al. 2021). Global gene expression analyses have revealed that the blue light photoreceptor ZTL up-regulates the expression of *PIF4* and PIF4-controlled genes including *YUC8*, thereby promoting hypocotyl growth at warm temperatures (Saitoh et al. 2021). In response to UV-B and warm temperatures, UVR8 inhibits PIF4 by first, together with COP1, decreasing the PIF4 protein level, and second, directly inhibiting PIF4 by way of the UV-B stabilized transcription factor HFR1. These molecular actions result in reduced PIF4 activity and attenuated thermomorphogenic response (Hayes et al. 2017).

Genetic studies have found that PIF1, PIF3, PIF4, PIF5 and PIF7 all contribute to the facilitation of thermomorphogenic growth (Chung et al. 2020; Fiorucci et al. 2020; Lee et al. 2021a). HECATEs (HECs) and PIFs form a negative feedback loop at the transcriptional level in response to warm temperatures. In parallel, HECs directly repress the activity of PIF4 toward target genes. Together, these regulatory events promote thermomorphogenesis (Lee et al. 2021a). PIF4 and PIF7 are likely dependent on each other in promoting thermomorphogenesis (Fiorucci et al. 2020). At elevated temperatures, the abundance of PIF7 accumulates rapidly due to an increase in translation (Chung et al. 2020). Similar to PIF4, PIF7 binds directly to the promoter regions of auxin biosynthesis and signaling genes to promote thermosensory growth (Chung et al. 2020; Fiorucci et al. 2020). In summary, various light signal and warm temperatures intersect and converge on PIFs to promote adaptive plant growth.

In addition to hypocotyl growth, warm temperatures also regulate the other physiological and developmental processes in plants such as stomatal development and leaf senescence through PIFs signaling. Elevated temperatures lead to the accumulation of PIF4 in stomatal precursors where it binds to the promoter of *SPEECHLESS* (*SPCH*) to repress its expression, thereby restricting stomatal development (Lau et al. 2018). Warm temperatures inactivate phyB and increase PIF4 abundance, which then directly associates with the promoter of positive aging regulator *ORESARA1* (*ORE1*) to activate its expression as well as abscisic acid (ABA) and ethylene signaling, subsequently promoting leaf senescence (Kim et al. 2020). Further, global transcriptomic analyses suggest that PIF4 and PIF5 facilitate warm temperature-triggered leaf senescence through multiple hormone signaling pathways (Li et al. 2021b). It will be essential and necessary to investigate the modes of action of distinct PIFs in the control of diverse light- and warm temperature-mediated biological processes in plants.

## COP1-HY5 signaling-mediated thermomorphogenic response

**Fig. 2:**
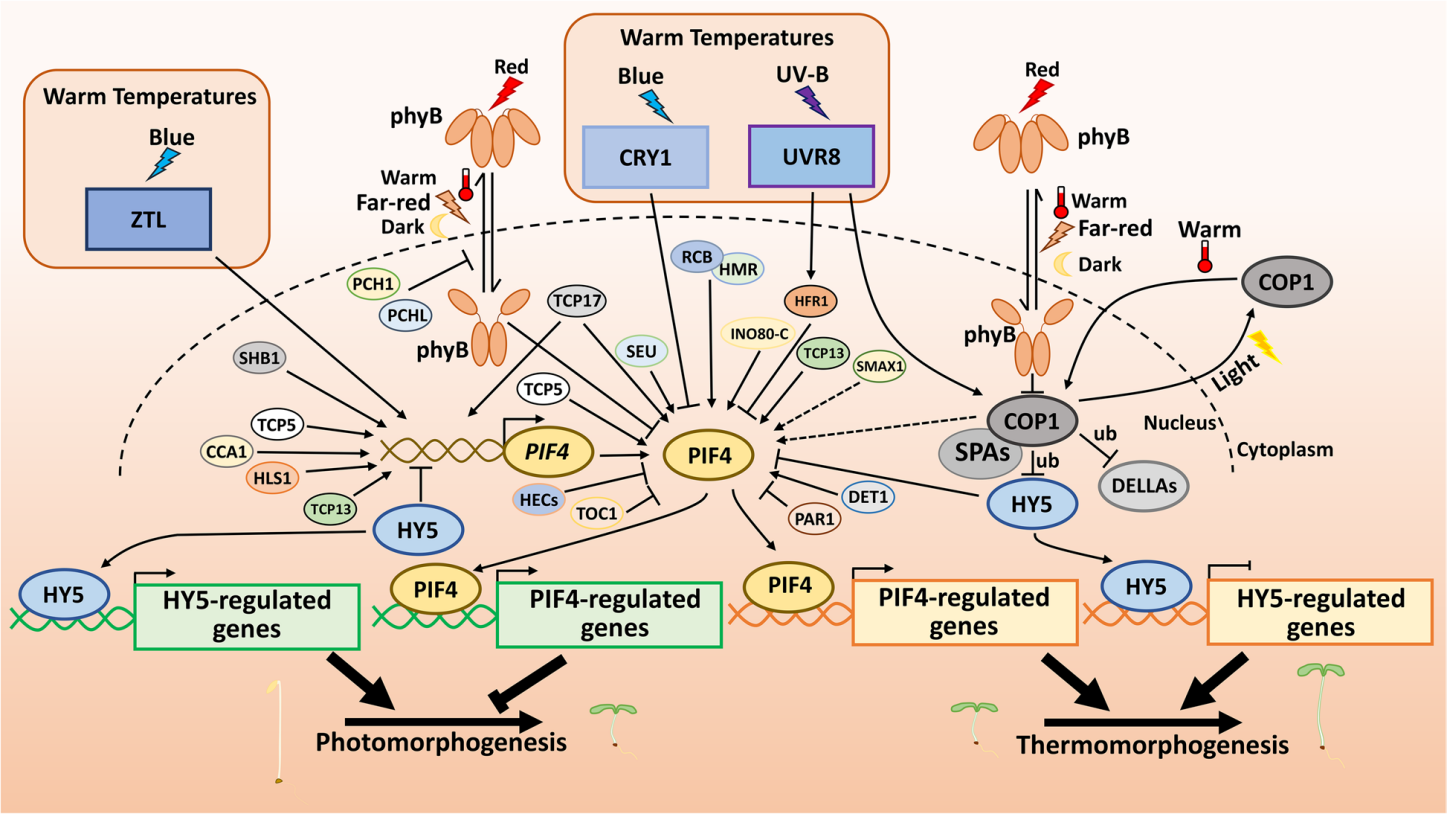
PIF4 and COP1-HY5 integrate light and warm temperature signaling. Warm temperatures trigger the conversion of phyB from active Pfr to inactive Pr form, thereby allowing the accumulation of PIF4. HLS1 SHB1 TCP5, TCP13 and TCP17 positively regulate the *PIF4* expression*,* while HY5 represses the expression of *PIF4* at the transcriptional level. CCA1, COP1-SPAs, DET1, HMR, IN080-C, RCB, SEU, SMAX1, TCP5, TCP13 and TCP17 enhance the PIF4 action, whereas HECs, HFR1, HY5, PAR1 and TOC1 and inhibit the PIF4 activity at the protein level. In blue light at warm temperatures, ZTL positively controls the transcription of *PIF4*, and CRY1 inhibits the PIF4 transcriptional activity. UVR8 not only inhibits the PIF4 activity trough HFR1, but also promotes the COP1 activity in response to UV-B and warm temperatures. Light induces the COP1 translocate from the nucleus to the cytoplasm, whereas warm temperatures have opposite effects on COP1 nuclecytoplamic partioning. COP1 destabilizes the HY5 and DELLA proteins through ubiquitinaiton at warm temperatures. ub represents ubiquitiation

Nucleocytoplasmic partitioning of COP1 is a key molecular strategy for modulating COP1-HY5 activity (von Arnim and Deng 1994; Hoecker 2017; Podolec and Ulm 2018; Han et al. 2020). Warm temperatures induce COP1 to translocate from the cytoplasm to the nucleus, where it facilitates the degradation of HY5 to promote hypocotyl growth and to repress anthocyanin accumulation (Park et al. 2017; Kim et al. 2017). Similarly, warm temperatures suppress seed germination by modulating the activity of the COP1-HY5 module (Chen et al. 2019). COP1 targets DELLA proteins, including RGA and GAI, for ubiquitination and subsequent degradation in response to warm temperatures (Blanco-Touriñán et al. 2020). COP1 and DE-ETIOLATED 1 (DET1), together with HY5, coordinate to transcriptionally regulate *PIF4* to promote thermosensory growth (Delker et al. 2014; Gangappa and Kumar 2017). In fact, DET1 and COP1 positively control PIF4 at both the transcriptional and protein levels. Furthermore, HY5 competes with PIF4 for binding to target sites to inhibit seedling growth in response to warm temperatures (Gangappa and Kumar 2017). A very recent work has shown that phys, PIFs, and HY5 all mediate root elongation at elevated temperatures. In root cells, HY5 directly controls the expression of numerous genes involved in the auxin and brassinosteroid signaling pathways, thus promoting root thermomorphogenesis (Gaillochet et al. 2020; Lee et al. 2021b). Collectively, COP1-HY5, together with other light signaling components, work synergistically to form a molecular regulatory network to allow for efficient and effective plant adaptation to changing light and warm temperature conditions (Fig. 2).

## ELF3-PIF4 signaling-mediated warm temperature response

**Fig. 3:**
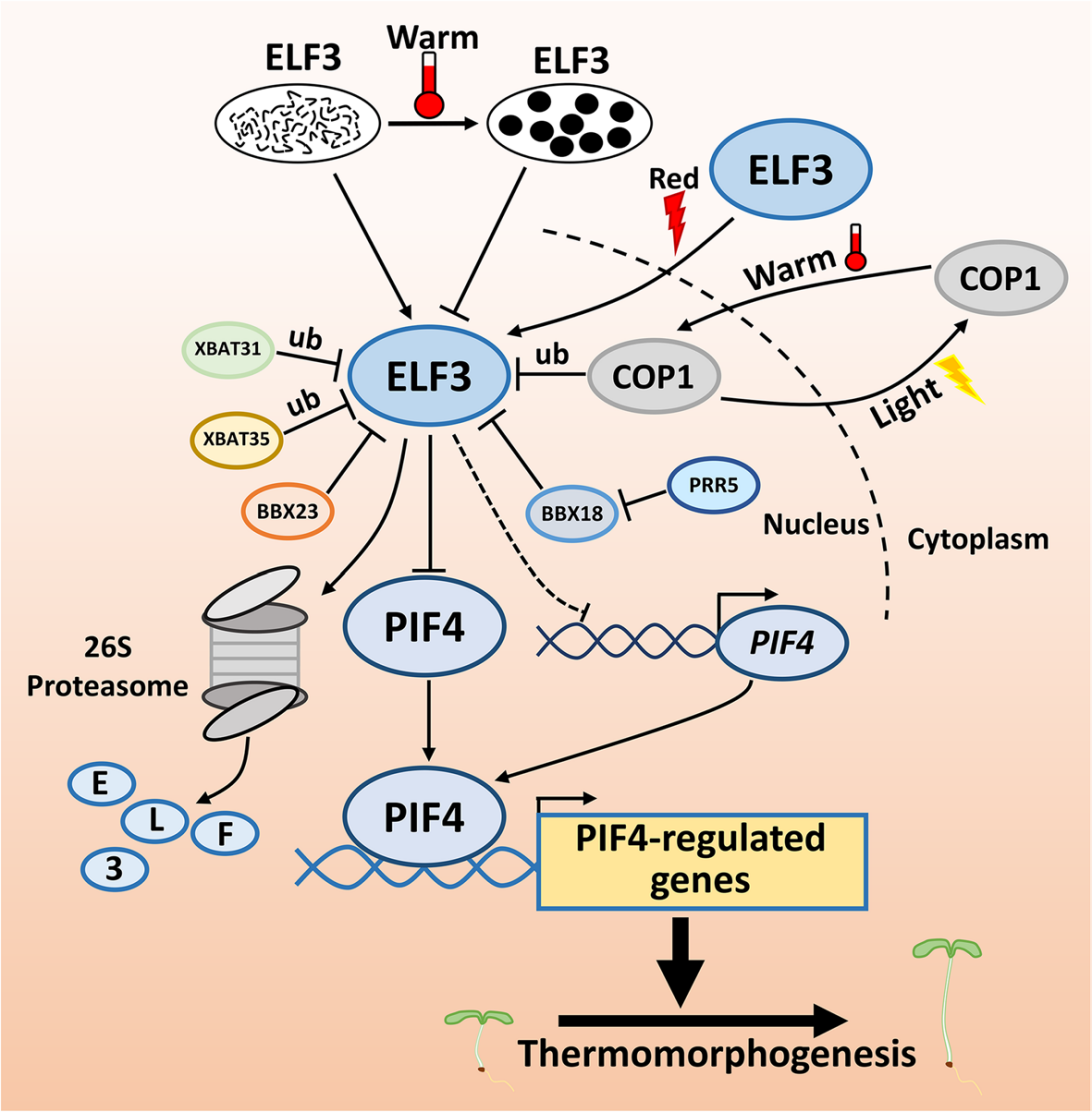
ELF3-PIF4-mediated thermomorphogenic response. Warm temperatures induce the ELF3 to form liquid droplets, thereby releasing the inhibition of PIF4 action. XBAT31 and XBAT35 target the ELF3 for ubiquitination and subsequent degradation. BBX18 and BBX23 facilitate the ELF3 degradation by recruiting ELF3 to XBAT31 and XBAT35. PIF4 regulates the expression of downstream target genes to promote thermomorphogenesis. ub represents ubiquitination

Light and circadian rhythm cues are integrated by the Evening Complex (EC) consisting of ELF3, ELF4, and LUX (Nusinow et al. 2011; Bu et al. 2021). Red light controls the cellular localization of ELF3, both dependent on and independent of phyB (Ronald et al. 2022). Additionally, ELF3 senses warm temperatures through its prion-like domain (PrD). Its closely interacting partner, ELF4, is required for stabilizing the temperature sensitivity of ELF3. Warm temperatures cause ELF3 PrD to form liquid droplets, a biologically inactive state (Jung et al. 2020), subsequently releasing the inhibitory effect of ELF3 on PIF4. In addition, ELF3 senses and stores daytime warm temperature cues and transmits this information to the *PIF4* promoter, consequently leading to the accumulation of PIF4 and hypocotyl growth during the night (Murcia et al. 2022). At warm temperatures, two E3 ubiquitin ligases, XB3 ORTHOLOG 1 IN *Arabidopsis Thaliana* (XBAT31) and XBAT35, directly target ELF3 for ubiquitination, thereby promoting its degradation via the 26S proteasome system. BBX18 facilitates the XBAT31 and XBAT35-mediated ubiquitination and degradation of ELF3 by recruiting ELF3 to these two E3 ligases. Further, genetic analyses have also shown that XBAT31 and XBAT35 positively control thermomorphogenesis (Zhang et al. 2021a, b). BBX18, together with BBX23, promote thermoresponsive hypocotyl growth through COP1- and PIF4-mediated signaling pathway at warm temperatures (Ding et al. 2018). Pseudo Response Regulators (PRRs) not only cooperate with EC complex to directly repress the expression of *PIF4* (Li et al. 2020b), but also repress PIF4 activity through a direct association (Zhu et al. 2016)*.* In the evening, BBX18 interacts with PRR5 to inhibit its repression of PIF4, accelerating thermoresponsive growth (Hwang et al. 2021). Taken together, ELF3-PIF4 may represent a key regulatory module that links the light and circadian clock to thermoresponsive growth in plants (Fig. 3).

## Concluding marks and future perspectives

Plants have evolved complex mechanistic frameworks which translate changing light and temperature stimuli into adaptational growth processes. Plants continuously sense and respond to fluctuating light and temperature cues, and light and temperature signaling share a set of components which work synergistically to regulate diverse physiological and developmental responses. Although tremendous efforts have been made to reveal the workings of the light and temperature signal transduction pathways, many detailed signaling interactions and interconnections remain obscure, especially in relation to daily and seasonal fluctuations at distinct latitudinal regions. Further research is required for gaining a comprehensive understanding of how plants sense and appropriately respond to dynamically changing light and temperature signals.

We know that phyB, ELF3 and PIF7 function as thermosensors through distinct regulatory mechanisms. Experimental evidence have shown that phyB senses the temperatures in a range from 10 °C to 30 °C, and ELF3 and PIF7 function at optimal growth temperatures (i.e. 22 °C) and warm temperatures (i.e. 28 °C) (Jung et al. 2016; Legris et al. 2016; Chung et al. 2020; Jung et al. 2020; Fiorucci et al. 2020). It would be of interest to determine whether these three thermosensors could sense cold signals (below 10 °C) as well. In particular, phyB acts as both a red-light photoreceptor and a thermosensor, and it has also been shown to mediate cold response (Jung et al. 2016; Legris et al. 2016; Jiang et al. 2020; Cheng et al. 2021). In addition, it has been demonstrated that the blue light photoreceptor PHOT acts as a cold sensor in the liverwort *Marchantia polymorpha* (Fujii et al. 2017). These intriguing facts suggest that light and both low and warm temperature signals are likely sensed and transduced by the same and/or similar signaling pathways. For example, both the PIFs and COP1-HY5 signaling modules act as regulatory hubs which integrate light as well as low and warm temperature signaling in order to appropriately modulate plant growth and development.

Considering that temperature is a physical signal (Zhu 2016), thus a variety of molecules in a plant cell likely could sense or response to this changing signal under natural conditions. As mentioned above, the photoreceptor phyB, the core component of EC ELF3 and transcription factor PIF7 function as thermosensory molecules through distinct modes of action. To further identify and characterize yet unknown temperature sensors or components of temperature signaling will help us to understand the regulatory network by which plants adjust to the surrounding changing temperatures. Light and temperature signals have profound effects on the various phytohormone biosynthesis and signal transduction in plants (Oh et al. 2012; Sun et al. 2012, 2013; de Wit et al. 2016; Fernández-Milmanda et al. 2020; Mao et al. 2020), suggesting that these external signaling pathways functionally link various internal phytohormone signaling to physiological and developmental output. It would be therefore of interest to dissect the detailed interplay between light, temperature and distinct hormone signaling in the regulation of plant morphogenesis at different developmental stages. The secondary metabolite biosynthesis and biomass accumulation are largely affected by the alternating of light and temperatures in different plant species. Multiple components of light and temperature signaling play critical roles in the regulation of these processes (Huang et al. 2019; Chen et al. 2021; Ge et al. 2022; Xiao et al. 2022). Thus, unraveling the exact molecular mechanisms by which light and temperatures coordinately regulate the various secondary metabolite biosynthesis and biomass production is a key fundamental issue. Although increasing studies have revealed the regulatory mechanisms underlying plants sense and response to light and temperature signals in controlled laboratory conditions (Ding and Yang 2022; Li et al. 2022; Qi et al. 2022), achieving a comprehensive understating of plants in response to dynamically changing light and temperatures in nature still remains to be a challenging task.

Much of our fundamental knowledge of light and temperature transduction was gained through the study of the model plant *Arabidopsis thaliana*. However, basic knowledge of light and temperature-mediated signaling in crop plants such as wheat, rape, rice, and soybean is grossly incomprehensive. Each plant species has its preferred growth environment, and diverse plant species may have evolved distinct, but overlapping, regulatory mechanisms to adapt to their preferred light and temperature regimes. For instance, wheat and rape are microthermic plants primary grown in spring or winter with low light intensities and temperatures, while rice and soybean are predominantly grown in summer and harvested in autumn, coinciding with high light intensities and temperatures. It is therefore essential and necessary to thoroughly unravel the complexity of regulatory mechanisms in diverse crops to ensure sustainable crop production in a changing environment.

## Data Availability

Not applicable.

## Abbreviations

CBF1: C-REPEAT/DRE BINDING FACTOR 1
CCA1: CIRCADIAN CLOCK ASSOCIATED 1
COP1: CONSTITUTIVE PHOTOMORPHOGENIC 1
COR27: COLD REGULATED GENE 27
COR28: COLD REGULATED GENE 28
CRY1: CRY1, CRYPTOCHROME 1
CRY2: CRYPTOCHROME 2
DET1: DE-ETIOLATED 1
EBF1: EIN3-BINDING F BOX PROTEIN 1
EBF2: EIN3-BINDING F BOX PROTEIN 2
ELF3: EARLY FLOWERING 3
ELF4: EARLY FLOWERING 4
HFR1: LONG HYPOCOTYL IN FAR-RED1
HLS1: HOOKLESS 1
HMR: HEMERA
HY5: ELONGATED HYPOCOTYL 5
KAI2: KARRIKIN INSENSITIVE 2
PAR1: PHYTOCHROME RAPIDLY REGULATED 1
PCH1: PHOTOPERIODIC CONTROL OF HYPOCOTYL 1
PCHL: PCH1-LIKE
phyA: PHYTOCHROME A
phyB: PHYTOCHROME B
PIF1: PHYTOCHROME INTERACTING FACTOR 1
PIF3: PHYTOCHROME INTERACTING FACTOR 3
PIF4: PHYTOCHROME INTERACTING FACTOR 4
PIF5: PHYTOCHROME INTERACTING FACTOR5
PIF7: PHYTOCHROME INTERACTING FACTOR 7
PHOT1: PHOTOTROPIN 1
PHOT2: PHOTOTROPIN2
RCB: REGULATOR OF CHLOROPLAST BIOGENESIS
SEU: SEUSS
SHB1: SHORT HYPOCOTYL UNDER BLUE1
SMAX1: SUPPRESSOR OF MAX2 1
TCP17: TEOSINTE BRANCHED 1/CYCLOIDEA/PCF 17
TOC1: TIMING OF CAB2 EXPRESSION 1
UVR8: UV-B RESISTANCE LOCUS 8
ZTL: ZEITLUPE
